# Case report: Checkpoint inhibitor pneumonitis with positive anti-melanoma differentiation-associated gene 5 antibodies in a patient with lung cancer

**DOI:** 10.3389/fimmu.2023.1309531

**Published:** 2024-01-12

**Authors:** Siqi Pan, Huaiya Xie, Luo Wang, Yuanzhuo Wang, Menglian Zou, Yan Xu, Xinlun Tian, Junping Fan, Jinglan Wang

**Affiliations:** ^1^ Department of Pulmonary and Critical Care Medicine, Peking Union Medical College Hospital, Chinese Academy of Medical Sciences & Peking Union Medical College, Beijing, China; ^2^ School of Clinical Medicine, Chinese Academy of Medical Sciences and Peking Union Medical College, Beijing, China; ^3^ Department of Internal Medicine, Peking Union Medical College Hospital, Chinese Academy of Medical Sciences & Peking Union Medical College, Beijing, China

**Keywords:** immune checkpoint inhibitors, immune-related adverse events, pneumonitis, rapidly progressive interstitial lung disease, anti-MDA5 antibodies

## Abstract

With the widespread use of immune checkpoint inhibitors to treat various cancers, pulmonary toxicity has become a topic of increasing concern. Anti-melanoma differentiation-associated gene 5 (anti-MDA5) antibodies are strongly associated with rapidly progressive interstitial lung disease (RP-ILD) in patients with clinically amyopathic dermatomyositis. However, anti-MDA5 antibody expression has not been reported in patients with immune-related adverse events. We present the case of a 74-year-old man with lung adenocarcinoma who developed RP-ILD after treatment with immune checkpoint inhibitors. Further investigation revealed multiple autoantibodies, including anti-MDA5 antibodies. He initially responded to systemic glucocorticoids, immunosuppressants, and tocilizumab but eventually died from worsening pneumomediastinum. This case is the first one to suggest that checkpoint inhibitor pneumonitis can present as RP-ILD with positive anti-MDA5 antibodies, which may be predictive of a poor prognosis.

## 1 Introduction

Immune checkpoint inhibitors (ICIs) can have various adverse effects, including checkpoint inhibitor pneumonitis (CIP), which can be life-threatening. Myositis and dermatomyositis (DM) are considered part of the spectrum of immune-related adverse events (irAEs). The relationship between anti-melanoma differentiation-associated gene 5 (anti-MDA5) antibodies and clinically amyopathic dermatomyositis was first described by Sato et al. in a Japanese cohort (1). Anti-MDA5 antibodies are considered markers of poor prognosis for rapidly progressive interstitial lung disease (RP-ILD) which refers to a course with measurable progression within a short period of time since onset of interstitial lung disease (ILD). However, anti-MDA5 antibodies have not been reported in patients with irAEs until now. Herein, we report the first case of a patient with CIP who tested positive for anti-MDA5 antibody.

## 2 Case presentation

**Figure 1:**
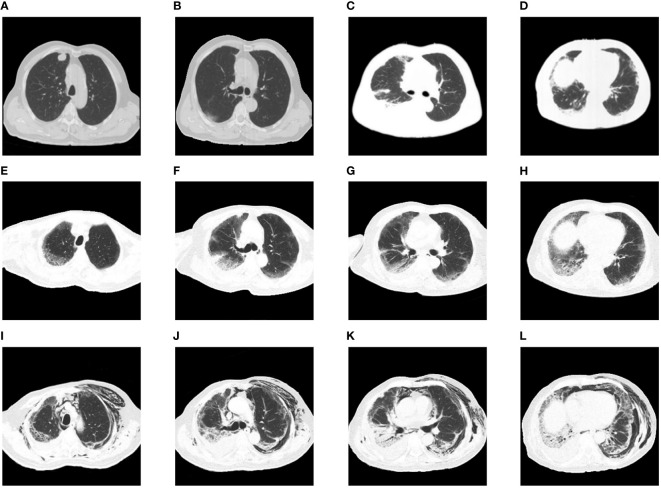
Chest images during the clinical episode. **(A, B)** The baseline chest CT before initiating therapy showed a nodule in the right upper lobe without any interstitial change. **(C, D)** Chest CT after onset of dyspnea showed new bilateral subpleural opacities, which were more severe in the right lung. **(E–H)** Chest CT on intubation day showed bilateral ground-glass opacities and consolidation with reticulation. **(I–L)** Chest CT after extubation revealed severe subcutaneous and mediastinal empysema, along with bilateral opacities consistent with interstitial pneumonitis.

A 74-year-old man was admitted to the Peking Union Medical College Hospital’s respiratory intensive care unit for a rash that lasted for one month and dyspnea that lasted for 2 weeks. He had been diagnosed with lung adenocarcinoma (**Figures 1A, B**) four months before admission. He had declined surgery due to severe comorbidities. Four weeks before admission, he was started on camrelizumab (200 mg, day 1), an ICI, combined with chemotherapy with pemetrexed (700 mg, day 1) and carboplatin (260 mg, day 1). He developed a pruritic maculopapular rash on his neck, chest wall, and sacrum. Two weeks before reporting to the hospital, he started experiencing shortness of breath on exertion, which gradually progressed. Chest computed tomography (CT) showed new bilateral subpleural opacities, which were more severe in the right lung (**Figures 1C, D**). CIP was suspected. Intravenous methylprednisolone 80 mg/day was administered for 5 days, tapered to 60 mg/day for 5 days, and maintained at 40 mg/day until admission. His rash improved but the dyspnea worsened. His medical history included hypertension, hyperlipidemia, cerebral infarction, and myocardial infarction. The patient had no history of ILD.

On physical examination, his body temperature was 36.5˚C, blood pressure was 147/56 mmHg, heart rate was 97 beats per minute, respiratory rate was 25 per minute, and oxygen saturation was 92% while he was receiving supplementary oxygen via a non-rebreathing mask at a flow rate of 12 liters per minute. Inspiratory tri-concave signs and scattered desquamated rashes were observed on inspection, and Velcro rales were heard bilaterally, but were more prominent in the right lung. Arterial blood gas (fraction of inspired oxygen was 70%) showed elevated pH (7.506), partial pressure of oxygen (76.6 mmHg; 83−108 mmHg), partial pressure of carbon dioxide (27 mmHg; 35−45 mmHg), and lactose concentration (2.3 mmol/L; 0.5−1.6 mmol/L). Elevated concentrations of C-reactive protein (69.8 mg/L; 0−3 mg/L), ferritin (1124 ng/ml; 24−336 ng/ml), and interleukin-6 (77 pg/ml; 0−5.9 pg/ml) were also documented. The rheumatology panel showed an elevated rheumatoid factor concentration of 57 IU/ml (0−20 IU/ml) and weak positivity for anti-nuclear antibody (1:160; normal range< 1:80 by ELISA), anti-mitochondrial M2 antibody (17; normal range< 15 by Western Blot), anti-MDA5 antibody (+; “+++” represents the strongest by Western Blot), and anti-Ro 52 antibody (+; “+++” represents the strongest by Western Blot). The other laboratory findings are presented in **Table 1**.

**Table 1:** Laboratory data.

Variable	Reference Range, Adults, This Hospital	On Admission
White-cell Count (10^9^/L)	3.50-9.50	7.46
Lymphocytes Count (10^9^/L)	0.80-4.00	0.33
Hemoglobin (g/L)	120-160	134
Platelet Count (10^9^/L)	100-350	251
Alanine Transferase (U/L)	9-50	41
Total Bilirubin (μmol/L)	5.1-22.2	7.3
Direct Bilirubin (μmol/L)	≤6.8	2.4
Albumin (g/L)	35-52	31
Urea (mmol/L)	2.78-7.14	7.18
Creatinine (μmol/L)	59-104	50
Lactate dehydrogenase (U/L)	0-250	437
Creatine Kinase (U/L)	24-195	27
Cardiac Troponin I (μg/L)	0-0.056	<0.017

Reference values are influenced by a wide range of factors, such as the patient group and the types of laboratory techniques. The ranges used at Peking Union Medical College Hospital are intended for adults without any health issues that might have an impact on the outcomes. They may not be appropriate for all patients.

**Figure 2:**
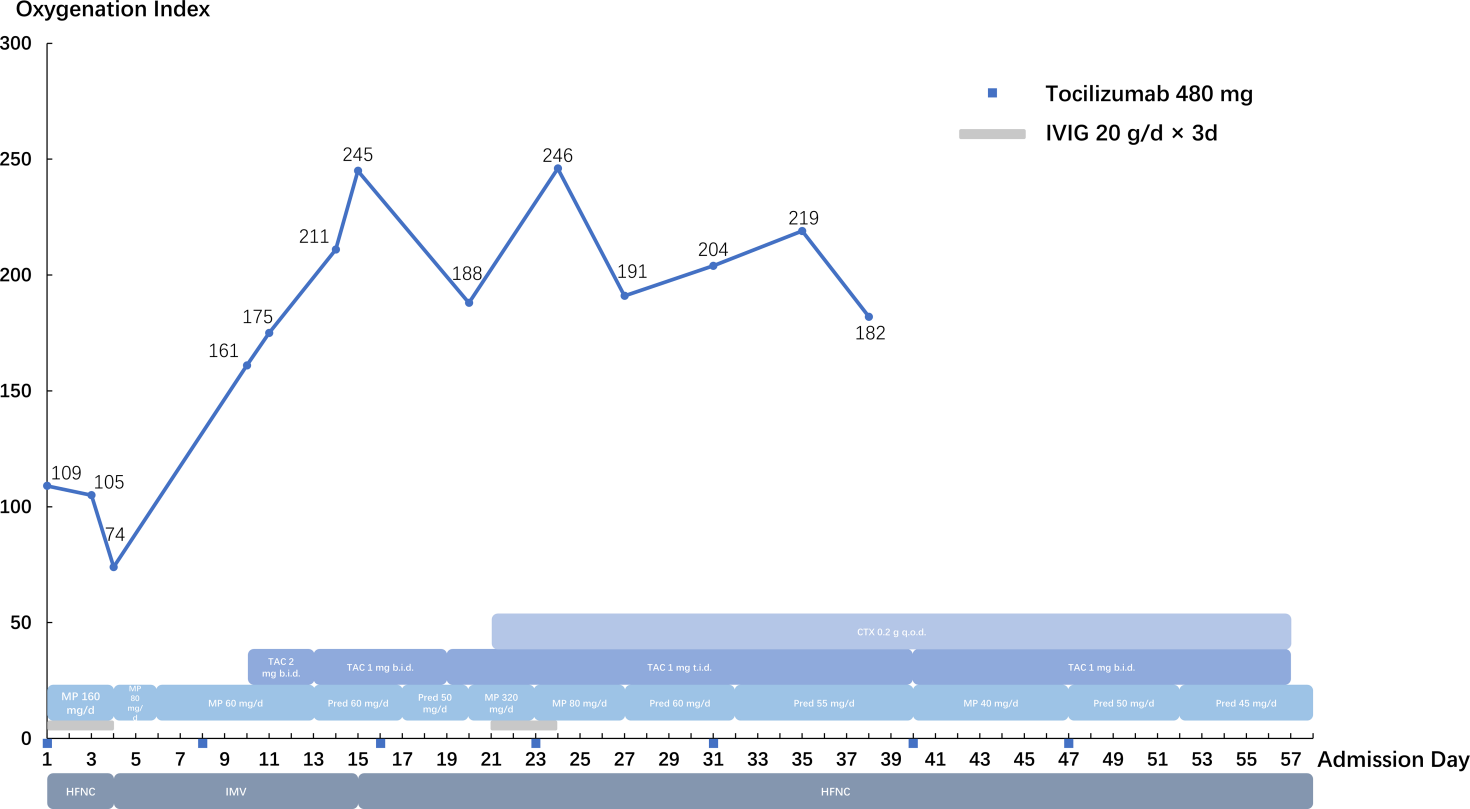
Summary of the clinical course. IVIG, intravenous immunoglobulin; HFNC, high-flow nasal cannula; IMV, invasive mechanical ventilation; MP, methylprednisolone; Pred, prednisone; TAC, tacrolimus; CTX, cyclophosphamide.

CIP (grade 4) was diagnosed, and the methylprednisolone dose was increased to 80 mg twice daily for 3 days before being tapered slowly. Tocilizumab (480 mg/week) was administered for 7 weeks, intravenous immunoglobulin was administered at 20 g/day for 3 days. Cyclophosphamide and tacrolimus were also administered. The patient was intubated and mechanically ventilated due to worsening respiratory distress. Chest CT (**Figures 1E–H**) showed bilateral ground-glass opacities and consolidation with reticulation. The patient improved and was successfully weaned from ventilation to a high-flow nasal cannula 11 days after intubation. However, he received a second round of intravenous methylprednisolone and intravenous immunoglobulin (20 g/day) due to exacerbation of his chest CT findings. Anti-MDA5 antibodies turned negative 20 days after the first report. His respiratory support level stabilized with the high-flow nasal cannula (flow rate of 30 L/min; fraction of inspired oxygen, 35−50%) until severe mediastinal and subcutaneous emphysema developed, which involved the neck and chest and extended to the scrotum and lower extremities (**Figures 1I–L**). Two months after admission, he strongly insisted on returning home. He died 2 days later due to severe dyspnea. The details of his treatment are summarized in **Figure 2**.

## 3 Discussion

ICIs are immunomodulatory antibodies that boost the immune system. Their main targets are programmed cell death receptor 1, programmed cell death ligand 1, and cytotoxic lymphocyte-associated antigen 4. ICIs have significantly improved the prognosis of patients with various advanced malignancies, including lung cancer. Immune-related adverse events (irAEs) are inflammatory responses caused by ICIs. They can involve the skin, gastrointestinal tract, endocrine system, lungs, and other organs (2). A study by Naidoo et al. showed that the overall incidence of CIP caused by anti-programmed cell death receptor 1 or programmed cell death ligand 1 treatment was 5%. The duration of treatment before pneumonitis onset varied with a median of 2.8 months (9 days to 19 months) (3). The clinical manifestations and imaging findings of CIP are nonspecific. Cough and dyspnea are the most common symptoms, but some patients may be asymptomatic. The signs and patterns on chest imaging include ground-glass opacity, consolidation, and diffuse alveolar damage. CIP is usually sensitive to glucocorticoid therapy (4). This patient developed a rash and pulmonary lesion soon after the administration of ICIs. Although his onset was rapid, immunotherapy-related skin and pulmonary toxicities should be considered first. Antineoplastic agent like pemetrexed can infrequently cause lung toxicities including ILD, with ground glass opacities the predominate CT pattern and good response to steroids, which should be differentiated in this case (5). However, pemetrexed is unlikely to cause the autoantibodies.

Anti-MDA5 antibodies are associated with clinically amyopathic dermatomyositis, which usually manifests as a characteristic rash and RP-ILD with high mortality. RP-ILD in anti-MDA5-positive DM is suggested to be defined as either worsening of dyspnea and CT progression within 1 month, or deterioration to respiratory failure within 3 months since respiratory symptom onset (6). According to the consensus of the 239th European Neuromuscular Centre meeting, DM can be diagnosed with the following criteria: typical DM-related rash, dermatopathological evidence of interface dermatitis, evidence of myositis, or positive DM-specific autoantibodies (7). Although this patient tested positive for anti-MDA5 antibodies, he did not have a typical DM-related rash or any evidence of myositis such as creatine kinase elevation; hence, DM could not be diagnosed. Moreover, he showed no signs of ILD on chest CT besides lung cancer at baseline. Anti-MDA5 and other autoantibodies before ICI therapy were not measured, which is a limitation of this report, but we disregarded them as a marker of newly developed or exacerbated anti-MDA5-positive DM. Additionally, the pathophysiology was different from that of typical anti-MDA5-positive DM in the following respects. First, the pulmonary lesion was more prominent on the tumor side. Second, the patient responded better to large doses of glucocorticoids and immunosuppressants and was successfully weaned from invasive mechanical ventilation, which is rare for patients with anti-MDA5-positive DM. Third, the titer of anti-MDA5 antibodies was mismatched with the severity of lung disease, and the patient tested negative for them immediately after therapy. A detailed comparison of anti-MDA5-positive DM, typical CIP, and CIP in this patient is shown in **Table 2**.

**Table 2:** Comparison of anti-MDA5-positive DM, typical CIP, and our patient’s condition.

	Anti-MDA5-Positive DM	Typical CIP	Our Patient’s condition
Rash Manifestation	Extensor articular erythema, periorbital erythema, typical DM-related rashes such as V sign, Gottron sign, and mechanic’s hands	Variable	Maculopapular rash
RP-ILD Manifestation	Always	Seldom	Yes
Mediastinal emphysema	Common	Seldom	Yes
Anti-MDA5 antibody and its titer	Positive, titer decreasing after effective treatment	No report so far	Positive, titer decreasing after effective treatment
Other autoantibodies	Anti-Ro 52 antibody	Seldom	ANA, AMA-M2, anti-Ro 52 antibody
Chest CT imaging manifestation	Interstitial changes in the bibasilar subpleural lung, manifesting as mass, reticulum, GGO	Variable	Bilateral subpleural GGO, the right side is prominent
Response to systemic glucocorticoids and immunosuppressants	Poor	Variable	Partial response
Prognosis	Poor	Depending on severity and grading	Poor

MDA5, melanoma differentiation-associated gene 5; DM, dermatomyositis; CIP, checkpoint inhibitor pneumonitis; RP-ILD, rapidly progressive interstitial lung disease; ANA, anti-nuclear antibody; AMA-M2, anti-mitochondrial M2 antibody; GGO, ground glass opacity.

ICIs can lead to various rheumatic irAEs such as arthritis and myositis, as well as presence of new autoantibodies. The incidence of ICI-induced myositis is approximately 0.6% (8). Ghosh et al. systematically reviewed the incidence of autoantibodies in patients with irAEs and found that 67 patients tested positive for myositis-associated antibodies, and 27% tested positive for at least one antibody, such as anti-Mi-2 antibody, anti-PM/Scl antibody, and anti-signal recognition particle (SRP) antibody (9). However, anti-MDA5 antibodies, which represent a type of myositis-associated antibodies, have not been previously reported after the administration of ICIs. In short, it is more reasonable to consider anti-MDA5 antibodies and other autoantibodies in this patient as byproducts of immunotherapy.

Anti-MDA5 antibodies are not found exclusively in patients with DM. Wang et al. reported that 48.2% of patients with coronavirus disease 2019 (COVID-19) tested positive for anti-MDA5 antibodies, and the non-survival group had a greater prevalence (10). Based on the shared features between this case and classical anti-MDA5-positive DM patients, we speculate that the anti-MDA5 antibodies may have partially accounted for the clinical characteristics justifying the use of immunosuppressive medication, and might be predictive of a poor prognosis. Tocilizumab, an interleukin-6 receptor monoclonal antibody, is licensed for the treatment of rheumatoid arthritis, systemic juvenile idiopathic arthritis, and COVID-19. It has been reported to improve the outcomes of patients with irAEs (11) and is utilized as salvage treatment for anti-MDA5-positive DM with RP-ILD (12). In our case, the administration of tocilizumab may have contributed to clinical remission.

## 4 Conclusion

We report the first case of a patient with CIP with anti-MDA5 antibody positivity, who transiently responded to treatment with glucocorticoids, immunosuppressants, and tocilizumab. However, he eventually died from dyspnea. Therefore, screening for anti-MDA5 antibodies is warranted in patients with CIP who present with RP-ILD.

## Data availability statement

The original contributions presented in the study are included in the article/supplementary material. Further inquiries can be directed to the corresponding authors.

## Ethics statement

Ethical approval was not required for the study involving humans in accordance with the local legislation and institutional requirements. Written informed consent to participate in this study was not required from the participants or the participants’ legal guardians/next of kin in accordance with the national legislation and the institutional requirements. Written informed consent was obtained from the individual(s) for the publication of any potentially identifiable images or data included in this article.

## Author contributions

SP: Conceptualization, Investigation, Writing – original draft, Writing – review & editing. HX: Data curation, Validation, Writing – review & editing. LW: Data curation, Validation, Writing – review & editing. YW: Validation, Writing – review & editing. MZ: Data curation, Resources, Writing – review & editing. YX: Writing – review & editing. XT: Supervision, Validation, Writing – review & editing. JF: Conceptualization, Funding acquisition, Resources, Supervision, Writing – review & editing. JW: Project administration, Supervision, Writing – review & editing.
